# Exploring specific prognostic biomarkers in triple-negative breast cancer

**DOI:** 10.1038/s41419-019-2043-x

**Published:** 2019-10-24

**Authors:** Chang Bao, Yunkun Lu, Jishun Chen, Danni Chen, Weiyang Lou, Bisha Ding, Liang Xu, Weimin Fan

**Affiliations:** 1 Program of Innovative Cancer Therapeutics, Division of Hepatobiliary and Pancreatic Surgery, Department of Surgery, First Affiliated Hospital, College of Medicine, Zhejiang University, Hangzhou, 310003 China; 2 Key Laboratory of Organ Transplantation, Hangzhou, 310003 China; 3 Key Laboratory of Combined Multi-organ Transplantation, Ministry of Public Health, Hangzhou, 310003 China; 4 Department of Cell Biology and Program in Molecular Cell Biology, College of Medicine, Zhejiang University, Hangzhou, 310003 China; 5 Clinical Research Center, First Affiliated Hospital of Zhejiang University College of Medicine, Hangzhou, 310000 China; 6 Department of Pathology and Laboratory Medicine, Medical University of South Carolina, Charleston, SC 29425 USA

**Keywords:** Breast cancer, Cell growth, Cell migration

## Abstract

Lacking of both prognostic biomarkers and therapeutic targets, triple-negative breast cancer (TNBC) underscores pivotal needs to uncover novel biomarkers and viable therapies. MicroRNAs have broad biological functions in cancers and may serve as ideal biomarkers. In this study, by data mining of the Cancer Genome Atlas database, we screened out 4 differentially-expressed microRNAs (DEmiRNAs) between TNBC and normal samples: miR-135b-5p, miR-9-3p, miR-135b-3p and miR-455-5p. They were specially correlated with the prognosis of TNBC but not non-TNBC. The weighted correlation network analysis (WGCNA) for potential target genes of 3 good prognosis-related DEmiRNAs (miR-135b-5p, miR-9-3p, miR-135b-3p) identified 4 hub genes with highly positive correlation with TNBC subtype: FOXC1, BCL11A, FAM171A1 and RGMA. The targeting relationships between miR-9-3p and FOXC1/FAM171A1, miR-135b-3p and RGMA were validated by dual-luciferase reporter assays. Importantly, the regulatory functions of 4 DEmiRNAs and 3 verified target genes on cell proliferation and migration were explored in TNBC cell lines. In conclusion, we shed lights on these 4 DEmiRNAs (miR-135b-5p, miR-9-3p, miR-135b-3p, miR-455-5p) and 3 hub genes (FOXC1, FAM171A1, RGMA) as specific prognostic biomarkers and promising therapeutic targets for TNBC.

## Introduction

Worldwide, breast cancer becomes the most commonly diagnosed malignance among women, accounting for 30% of all new cancer diagnoses in 2019^1^. Despite important advances in early detection and research development, breast cancer remains a major health problem affecting women. In 2019, breast cancer alone accounts for 15% of all cancer deaths, inferior to lung and bronchus^1^, and the disease burden becomes more severe in young women aged < 45 years old^2^. Comprehensive gene expression profiling has identified 4 major molecular subtypes of breast cancer, including luminal A, luminal B, HER2 positive and TNBC, which are characterized by specific biological properties, morphological patterns and, more importantly, distinct clinical process and prognosis^3,4^. The most aggressive subtype is TNBC, lacking of estrogen receptor (ER), progesterone receptor (PR) and human epidermal growth factor receptor 2 (HER2), accounting for 15–20% of all breast cancers^5^. Unfortunately, due to the devoid of early detection biomarkers and clear therapeutic targets, TNBC patients are often diagnosed late with a high histological grade, and do not benefit from hormonal or targeted therapies^6^. As a result, patients with TNBC usually suffer high risks of metastasis and distal recurrence, and have poor prognosis with shortened disease-free survival (DFS) and overall survival (OS)^7,8^. Hence, as a serious clinical challenge, TNBC calls for urgent needs of developing novel prognostic biomarkers and therapeutic targets.

MicroRNAs (miRNAs) are a group of endogenous non-coding RNAs, which are single-stranded and ~21 nucleotides (~21 nt) in length. They modulate genes expression on post-transcriptional level by directly binding to the 3′untranslated regions (3′-UTRs) of multiple target genes^9^. Numerous miRNAs have been reported to regulate diverse biological processes, such as proliferation, cell cycle control, metastasis, apoptosis and differentiation, acting as tumor suppressor genes or oncogenes^10–12^. Accumulating studies have suggested that miRNAs are frequently dysregulated in tumors compared with normal tissues, resulting in aberrant expression of target genes or proteins and cancer progression^13^. Recently, DEmiRNAs identified in TNBC have been found to be associated with the aggressive phenotype^14,15^. More importantly, emerging evidences have shown miRNAs could be applied to clinic as diagnostic/prognostic biomarkers and therapeutic strategies for breast cancer^16–18^. Overall, identification of novel biomarkers for TNBC is currently a high priority, and miRNAs as potential biomarkers, require better understanding of their roles and mechanisms in TNBC. However, due to limited patient number and distinct sequencing platforms, most of studies lack a normalized standard. The Cancer Genome Atlas (TCGA), as a landmark cancer genomics database, comprises multiple levels of tumor data, including genomic, transcriptomic, proteomic, epigenetic and clinical data^19^. This publicly available database provides over 20,000 primary tumor and matched normal samples spanning 33 cancer types. Researches based on this database have already led to improvements of diagnosis, prevention and treatment for cancers^20–22^.

In this study, we screened out 4 candidate DEmiRNAs (miR-135b-5p, miR-9-3p, miR-135b-3p, miR-455-5p) that were significantly associated with the survival of TNBC cohort but not non-TNBC cohort. Moreover, we identified 4 hub genes (FOXC1, BCL11A, FAM171A1, RGMA) which showed highly positive correlation with TNBC subtype. Importantly, we provided evidences that miR-9-3p targeted FOXC1/FAM171A1, and miR-135b-3p targeted RGMA in TNBC cells. Furthermore, the regulatory effects of 4 DEmiRNAs and 3 verified hub genes on cell proliferation and migration were confirmed. Therefore, our findings might provide advancements in the ongoing effort to develop specific prognostic biomarkers and potential therapeutic targets for TNBC.

## Materials and methods

### Screening for DEmiRNAs

The miRNA expression data of breast cancer (BRCA) samples measured by Illumina-Hiseq were retrieved from TCGA database (https://genome-cancer.ucsc.edu/) updated by the end of March 31, 2017, including 2238 miRNAs obtained from 749 tumor samples (81 TNBC and 668 non-TNBC samples) and 76 normal samples. Data were normalized and then conducted miRNA differential expression analysis using R package limma^23^ from the bioconductor project. The DEmiRNAs were respectively identified in TNBC and non-TNBC samples, both compared with normal BRCA samples. Adjust *P* value < 0.05 and |log_2_ fold change (log_2_FC)| > 1 were set as the thresholds for identifying DEmiRNAs.

### Weighted gene co-expression analysis (WGCNA)

A co-expression network was built according to the protocols of R package WGCNA^24^ in R environment. Briefly, we created a matrix of pairwise Pearson correlation coefficients to measure the gene-gene similarity across the samples. Then we used a power adjacency function in this R package to transform the similarity matrix into an adjacency matrix which encodes the connection strengths of pairwise nodes in the network^25^. The power β = 5 was chosen based on the scale-free topology criterion to determine a scale-free topology index (R2) of 0.84 for TCGA cohorts. Then we used the Topological Overlap Measure (TOM) that is average linkage hierarchical clustering with a dissimilarity measure to detect gene modules. This measure is a robust measure of network interconnectedness, which represents the overlap observed between shared neighbors^26^. Modules were regarded as branches of the dendrogram, and were cut by the Dynamic Tree-Cut algorithm^27^. Meanwhile we calculated module eigengene to represent and summarize each module by measuring the first principal component of a given module.

Next, we used Module-Trait Relationships (MTRs) from WGCNA package to determine the significant correlation between module eigengene and BRCA traits (subtypes) classified by TCGA database. For intramodular analysis, we evaluated the Gene Significance (GS) and Module Membership (MM), the latter of which was also called eigengene-based connectivity (kME). GS is the absolute value of the correlation between a specific gene and a trait; MM is the correlation between module eigengene and gene expression profile. By analysis of GS and MM, we identifed genes that showed significant MM and high GS for TNBC subtype. The network diagrams were depicted in Cytoscape software.

### GO and KEGG pathway enrichment analysis

The visualization of GO and KEGG pathway enrichment analysis for green module genes used R package clusterProfiler^28^ from the bioconductor project. Adjust *P* value < 0.05 was considered as statistically significant.

### Breast cancer cell lines

The human normal breast epithelial cell line (HBL-100), 5 TNBC cell lines (MDA-MB-231, BCap37, Hs 578 T, BT-549, HCC1937) and the non-TNBC cell line (MCF-7) were purchased from the Cell Bank of the Chinese Scientific Academy. HBL-100, BCap37, BT-549, HCC1937 and MCF-7 were cultured in Roswell Park Memorial Institute (RPMI) 1640 medium (Gibco, 31800105, Life technologies, Carlsbad, CA, USA) with 10% fetal bovine serum (FBS; Biological Industries, 04-0101-1, Cromwell, CT, USA). MDA-MB-231 were cultured in Leibovitz’s L-15 medium (Gibco, 11415114) with 10% FBS. Hs 578 T were cultured in Dulbecco’s Modified Eagle’s (DMEM) Medium (ATCC® 30-2002™), with 10% FBS. All cells were incubated at 37 °C with 5% CO_2_ in a water-jacketed incubator (Thermo Scientific, Waltham, MA, USA). The cell culture medium was changed every two days, and experiments were initiated when cells showed logarithmic growth at 70–80% confluence.

### Cell transfection

The mimics of miR-135b-5p (135b-5p), miR-9-3p (9-3p), miR-135b-3p (135b-3p), miR-455-5p (455-5p), and the inhibitors of miR-455-5p (in-455-5p) were purchased from Ribobio (Guangzhou, China). The siRNAs of FOXC1, FAM171A1 and RGMA were purchased from GenePharma (Shanghai, China). Above miRNA mimics/inhibitors and siRNAs were transfected into cells using Lipofectamine^TM^ 3000 according to the manufacturer’s instructions. After 12 h of transfection, cells were changed fresh cell culture medium for following experiments.

### MTT cell viability assay

Cells were evenly added into 96-well plates with 2 × 10^3^ cells per well. After 12 h, each column was transfected with specific reagents. Cells were incubated for various durations as indicated after which 15 μl of MTT (5 mg/ml) solution were added into each well. After additional 4 h in the incubator, the absorbance of each well was measured under 570 nm wavelength. Cell growth curves were depicted in Graphpad Prism 7 software.

### Quantitative real-time PCR (qRT-PCR)

Total RNA of cells was extracted using RNAiso plus Reagent (TaKaRa biotechnology, 9109, Kusatsu, Japan), and then was reverse transcribed into complementary DNA (cDNA) using PrimeScript RT Reagent kit (TaKaRa biotechnology, RR037A). qRT-PCR was performed by the Roche LightCycler480 II Real-time PCR Detection System using TB Green Premix Ex Taq^TM^ (TaKaRa biotechnology, RR420A). Quantification of miRNAs was performed with stem-loop RT-PCR, using U6 as internal reference. The gene expression was relative to GAPDH. All reactions were set triplicate duplications and were calculated by the comparative threshold method (2^–ΔΔCt^). The sequences of primers for qRT-PCR are listed in Table S2.

### Colony formation assay

Cells were evenly plated into 6-well plates (300 cells per well). After 12 h, miRNA mimics were respectively transfected into BCap37 cells. After 12 h of transfection, the cell culture medium was changed every two days and cells were transfected once again on the 6th day. Until the 12th day, cells were fixed with 4% Paraformaldehyde (Dalian Meilun Biotechnology, MA0192, China) for 15 min. The fixed cells were washed three times by PBS, and then were dyed with 0.1% Crystal violet staining solution for 30 min. Finally, the viable colonies with >50 cells were counted.

### Dual-luciferase reporter assay

Through TargetScan database, miR-9-3p was predicted one binding site with 3′ -UTRs of FOXC1, BCL11A or FAM171A1, and miR-135b-3p was predicted one binding site with 3′ -UTRs of RGMA (Fig. S3A). To construct wide type (wt) reporter plasmids, around 200 bp of sequences upstream and downstream of the binding site were amplified and inserted between NotI and XhoI of psiCHECK-2 (Promega, USA) vector. Then the binding sites were mutant to construct mutant type (mut) vectors. For dual-luciferase reporter assay, MDA-MB-231 cells were evenly distributed into 96-well plate with 8 × 10^3^ cells per well. After 12 h, miRNA mimics and wt/mut-reporter vectors were co-transfected for another 24 h incubation. Luciferase activity was measured by the Reporter Assay System Kit (Promega, 017319). Firefly luciferase activity was normalized to Renilla luciferase. The sequences of primers used to construct wt/mut reporter plasmids are listed in Table S2.

### Wound healing assay

MDA-MB-231 cells were plated in 6-well plates with 2 × 10^5^ cells per well. 12 h later, cells were transfected with specific miRNA mimics, inhibitors or siRNAs. When cells grew to 100% confluence, a micropipette tip was used to make a straight wound in each well. Photographs were taken by a microscopy at 0 h, 24 h or 48 h after wounding.

### Transwell migration assay

Cells were initially plated in 12-well plates for 48 h of transfection with specific reagents. The upper inserts of 24-well transwell chambers (Corning, USA) were added 2 × 10^4^ cells re-suspended in 0.2 ml serum-free medium. The lower compartments were added 0.6 ml medium with 10% FBS as chemoattractant. After 24 h of incubation, cells on the upper surface of membrane were gently removed using a cotton bud, and cells on the lower surface were fixed with 4% Paraformaldehyde (Dalian Meilun Biotechnology, MA0192, China) for 15 min. Next, the fixed cells were washed three times by PBS, and were stained with 0.1% Crystal violet staining solution for 30 min. Five random fields of each insert were photographed and counted under a light microscope (Olympus, Japan).

### Western blotting

Proteins were extracted from cells, and were measured concentrations by a BCA protein assay kit (Beyotime Biotec, China). Protein samples were fractionated using 8 or 10% SDS-PAGE gels and then transferred to PVDF membranes (Millipore, NY, USA). After 1 h blocking with 5% non-fat milk at room temperature, membranes were incubated at 4 °C for 12 h with rabbit anti-human primary antibodies: FOXC1 (Abcam, ab227977, 1:1000), FAM171 A1 (GeneTex, GTX120226, 1:1000), RGMA (Abcam, ab169761, 1:10000); GAPDH (Diagbio, db106, 1:2000) and Tubulin (ABclonal, AC015, 1:2000) were used as endogenous controls. The proteins were detected by ECL detection solution (Thermo Scientific™) and analyzed by Image Lab software (Bio-Rad).

### Statistical analysis

All experiments were performed at least three times. Data were shown as mean ± standard deviation (SD). Two-tailed Student’s t-test was used to evaluate differences between two groups of data. The Kaplan–Meier method and log-rank test was used to evaluate the correlation between miRNA expression and patient survival. The WGCNA method was analyzed by Pearson correlation analysis. The Kruskal-wallis test was used to evaluate the gene expression in TNBC, non-TNBC and normal cohorts from TCGA database. (*P < 0.05; **P < 0.01; ***P < 0.001; ****P < 0.0001. *P* values < 0.05 were considered statistically significant).

## Result

### MiR-135b-5p, miR-9-3p, miR-135b-3p, miR-455-5p are specially correlated with the prognosis of TNBC

**Fig. 1:**
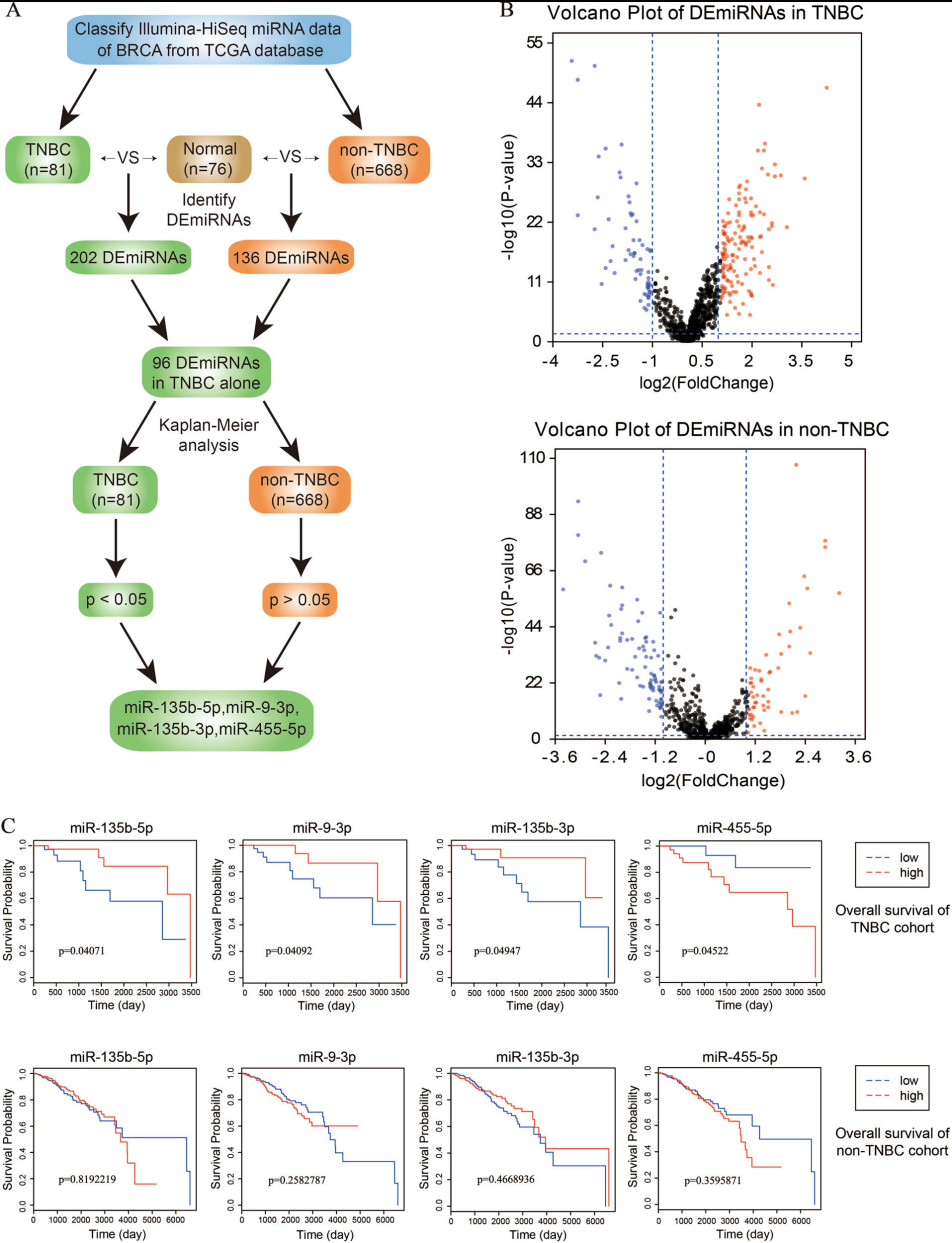
Identification of specific DEmiRNAs related to prognosis of TNBC alone. **a** The workflow for identification of DEmiRNAs specifically associated with the prognosis of TNBC. **b** The volcano plots of DEmiRNAs in TNBC (202 miRNAs) and non-TNBC (136 miRNAs) cohort. The red dots and blue dots respectively represent upregulated (FC > 2) and downregulated (FC < 0.5) DEmiRNAs with statistical significance. **c** The Kaplan–Meier analysis of 4 candidate DEmiRNAs in TNBC (*p* < 0.05) and non-TNBC (*p* > 0.05) cohort

As the workflow shown in Fig. 1a, a large-scale miRNAs expression data of BRCA samples were initially downloaded from TCGA database. By comparison with normal samples (*n* = 76), 202 DEmiRNAs (138 upregulated and 64 downregulated miRNAs) were detected in TNBC samples (*n* = 81), and 136 DEmiRNAs (54 upregulated and 82 downregulated miRNAs) were found in non-TNBC samples (*n* = 668) (Fig. 1a, b). A total of 96 DEmiRNAs in TNBC but not non-TNBC cohort were filtered out. In order to find out DEmiRNAs specially associated with the prognosis of TNBC, 96 DEmiRNAs were respectively performed Kaplan–Meier analyses of overall survival (OS) in TNBC and non-TNBC cohort. Finally, 4 candidate DEmiRNAs (miR-135b-5p, miR-9-3p, miR-135b-3p, miR-455-5p) were obtained owing to their significant correlation with the survival of TNBC alone (Fig. 1c). To be specific, high expression of miR-135b-5p, miR-9-3p, and miR-135b-3p showed good prognosis, whereas high expression of miR-455-5p exhibited poor prognosis of TNBC patients from TCGA database.

### The proliferation and migration of TNBC cell lines are inhibited by miR-135b-5p, miR-9-3p and miR-135b-3p, whereas promoted by miR-455-5p

**Fig. 2:**
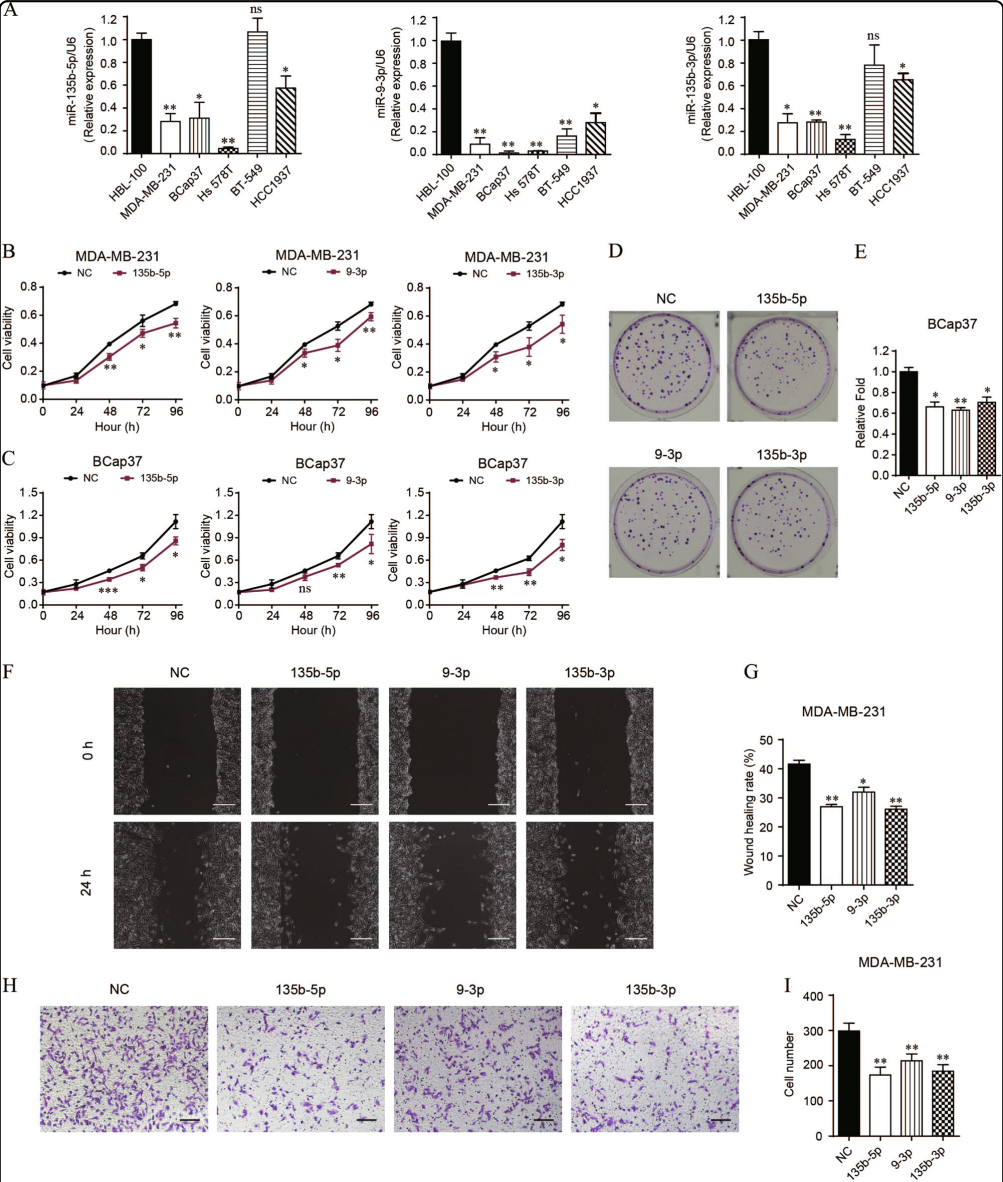
The proliferation and migration of TNBC cells are suppressed by miR-135b-5p, miR-9-3p and miR-135b-3p. **a** The expression levels of miR-135b-5p, miR-9-3p and miR-135b-3p were measured in 5 TNBC cell lines (MDA-MB-231, BCap37, Hs 578 T, BT-549, HCC1937) and a normal breast cell line (HBL-100). **b**, **c** The cell growth curves were depicted in MDA-MB-231 and BCap37 cells transfected with mimics of NC (NC), miR-135b-5p (135b-5p), miR-9-3p (9-3p) or miR-135b-3p (135b-3p), respectively. **d**, **e** The colony formation assay was performed in BCap37 cells transfected with specific miRNA mimics. **f**, **g** 24 h wound healing rates were measured in MDA-MB-231 cells transfected with specific miRNA mimics. Scale bars: 200 μm. **h**, **i** The transwell migration assays further confirmed the effects of 3 miRNAs on cell metastasis. Scale bars: 200 μm. Bars indicate the mean ± SD of three independent replicates. *P < 0.05, **P < 0.01, ***P < 0.001

We initially measured the expression of 4 DEmiRNAs in 5 TNBC cell lines (MDA-MB-231, BCap37, Hs 578 T, BT-549, HCC1937), compared with a human normal breast epithelial cell line (HBL-100). The results showed that miR-135b-5p/miR-135b-3p notably downregulated in 4 TNBC cell lines except for BT-549 (no significance), and miR-9-3p significantly downregulated in all 5 TNBC cell lines (Fig. 2a). However, the expression level of miR-455-5p was markedly upregulated in MDA-MB-231, Hs 578T, and BT-549 cell lines, but obviously decreased in BCap37 and HCC1937 cell lines (Fig. S2A). Among these TNBC cell lines, MDA-MB-231 and BCap37 were more suitable for functional assays. Thus we transfected MDA-MB-231 and BCap37 with mimics of miR-135b-5p, miR-9-3p or miR-135b-3p (Fig. S1A–C). However, considering that miR-455-5p upregulated in MDA-MB-231 whereas downregulated in BCap37, we knockdown it in MDA-MB-231 but overexpressed it in BCap37 to perform functional studies, respectively (Fig. S1D, E). As a result, miR-135b-5p, miR-9-3p and miR-135b-3p obviously decreased the cell viabilities of two TNBC cell lines (Fig. 2b, c), whereas miR-455-5p promoted the cell viabilities of BCap37 cells (Fig. S2B). In turn, knockdown of miR-455-5p in MDA-MB-231 cells showed a significant reduction of cell viabilities (Fig. S2C). Moreover, the colony formation assays were performed only in BCap37 since this cell line presented tight cell growth, whereas MDA-MB-231 showed dispersive cell growth (Fig. S2D). The results further confirmed that miR-135b-5p, miR-9-3p and miR-135b-3p significantly inhibited cell clonogenicity (Fig. 2d, e), while miR-455-5p markedly increased the clonogenicity (Fig. S2E, F). Furthermore, MDA-MB-231 was employed for cell migration assays owing to its high-metastatic behavior. The 24 h wound healing rates were significantly decreased by miR-135b-5p, miR-9-3p and miR-135b-3p (Fig. 2f, g), and the following transwell migration assays further confirmed they could obviously attenuate cell migration (Fig. 2h, i). In addition, the cell migration could be markedly suppressed by knockdown of miR-455-5p in MDA-MB-231 cells (Fig. S2G–J). Overall, we validated that the proliferation and migration of TNBC cell lines were inhibited by miR-135b-5p, miR-9-3p and miR-135b-3p, whereas promoted by miR-455-5p.

### Key gene module relevant to TNBC subtype was identified via WCGNA

**Fig. 3:**
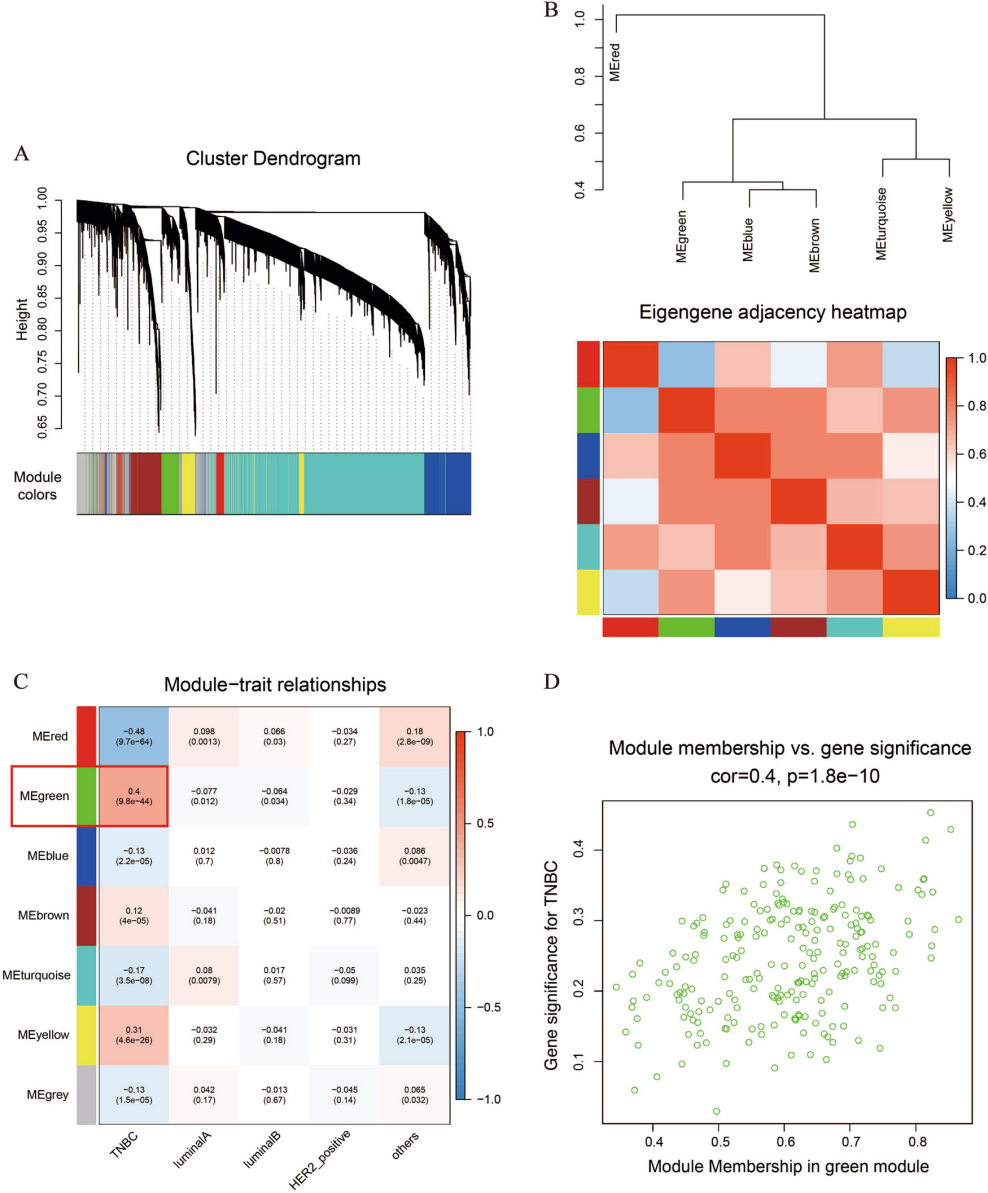
The WGCNA for potential target genes of miR-135b-5p, miR-9-3p and miR-135b-3p. **a** The predicted target genes of 3 good prognosis-related DEmiRNAs (miR-135b-5p, miR-9-3p, miR-135b-3p) were divided into 7 modules in the cluster dendrogram. **b** The eigengene of each colored module were calculated and established an adjacency matrix. **c** The BRCA samples form TCGA database were classified as 5 subtypes: TNBC (*n* = 115), luminal A (*n* = 361), luminal B (*n* = 98), HER2-positive (*n* = 37) and others (*n* = 477). The Module-Trait Relationships (MTRs) between module eigengenes (row) and BRCA subtypes (column). The green module highlighted in a red box showed the highest correlation with TNBC subtype. **d** Correlation analysis between module membership (MM) and gene significance (GS) for each gene in the green module

In order to better understanding the mechanisms of prognosis-related DEmiRNAs in TNBC, we conducted the weighted gene co-expression analysis (WGCNA) for their potential target genes. The WGCNA is a robust method to find out gene clusters with significant correlation with specific traits. We respectively conducted WGCNA for predicted target genes of 3 good prognosis-related DEmiRNAs (miR-135b-5p, miR-9-3p, miR-135b-3p) and that of the poor prognosis-related DEmiRNA (miR-455-5p). A total of 6013 target genes were predicted by TargetScan database^29^ for miR-135b-5p, miR-9-3p and miR-135b-3p, and 1089 BRCA samples from TCGA database were employed for constructing the gene co-expression network. Initially, highly co-expressed genes were detected as 6 colored modules by the Topological Overlap Measure (TOM), and the gray module represents background genes (Fig. 3a). Meanwhile, the eigengene was calculated as the representative for each module. Then the Pearson correlation coefficients were calculated to evaluate the agreement of pairwise modules (Fig. 3b). Through the method of Module-Trait Relationships (MTRs), eigengenes of 7 modules were respectively evaluated relevance with 5 BRCA subtypes classified by TCGA samples. Above all, the green module showed the highest positive correlation with TNBC, including 236 genes (Fig. 3c, Table S1). As shown in Fig. 3d, the positive correlation (*r* = 0.4; *p* = 1.8e-10) between module membership (MM) and gene significance (GS) of each gene in the green module was evident. However, due to the limited amount of potential target genes for miR-455-5p, no gene clusters were found to show significant correlation with TNBC subtype. Overall, by conducting WGCNA, the green module was identified as key gene cluster with highly positive correlation with TNBC.

### FOXC1, BCL11A, FAM171A1 and RGMA are identified as 4 hub genes with the highest correlation with TNBC

**Fig. 4:**
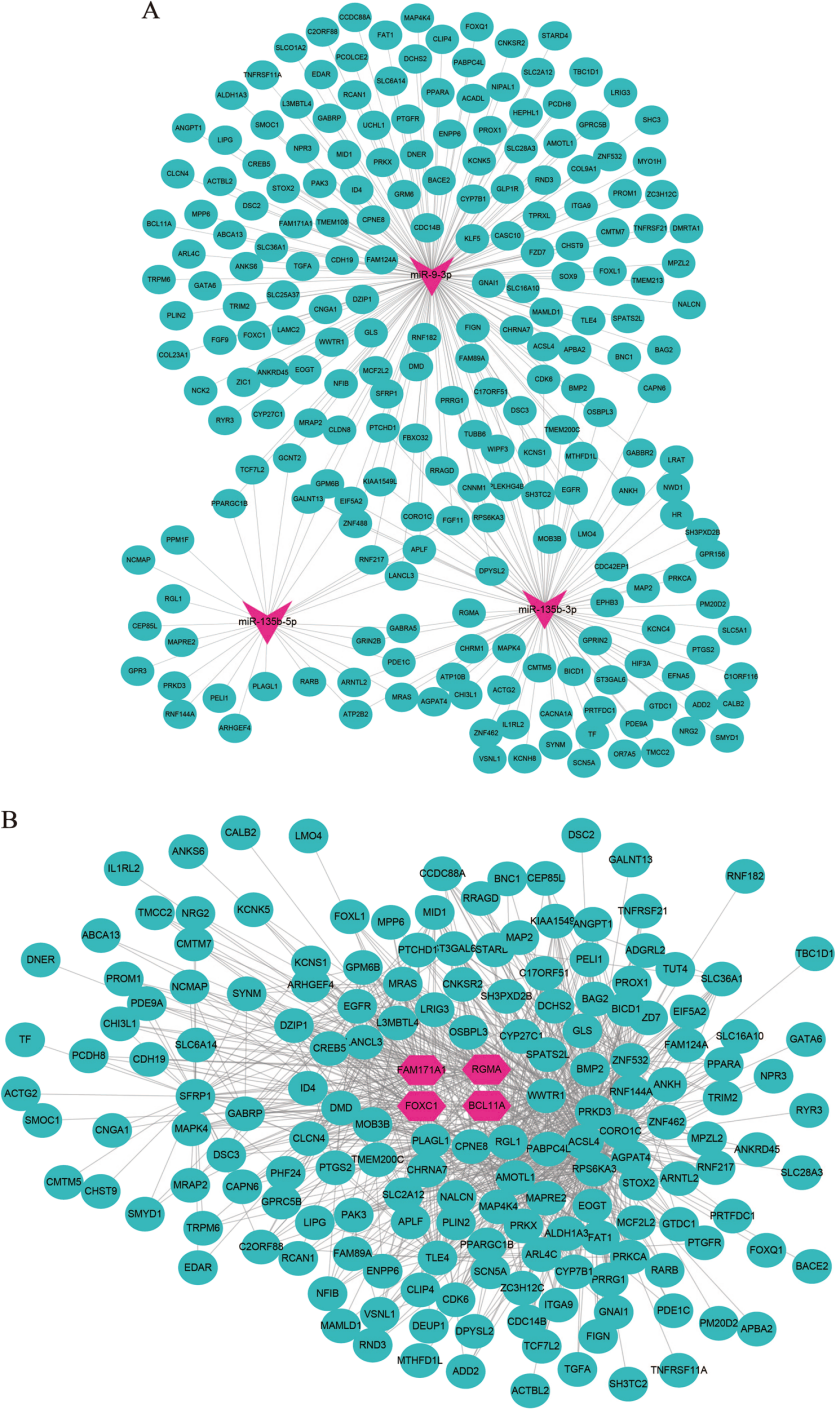
Identification of potential biological roles and hub genes for green module. **a** The network of potential targeting between miR-135b-5p/miR9-3p/miR-135b-3p and genes in the green module. **b** The co-expression network of genes in the green module (edge weight > 0.05). 4 hub genes were highlighted in red

In order to better understand the biological features and significances of genes in green module, the enrichment analyses of GO items and KEGG pathways were performed. The GO analysis (Fig. S3A) showed genes were enriched in positive regulation of epithelial cell proliferation, gland development, positive regulation of epithelial to mesenchymal transition, etc. The following KEGG pathway analysis (Fig. S3B) indicated that the green module genes were enriched in multiple classic cancer-related pathways, including ErbB, Ras and Rap1 signaling pathway. The network depicted in Fig. 4a indicated the potential targeting relationships between green module genes and 3 good prognosis-related DEmiRNAs (miR-135b-5p, miR-9-3p and miR-135b-3p). Moreover, to visualize gene interactions of the green module, we portrayed another network diagram (Fig. 4b). In the center of this network, 4 genes (FOXC1, BCL11A, FAM171A1, RGMA), highlighted in red, were identified as 4 hub genes with the highest correlation with TNBC (MM > 0.7, GS > 0.4).

### FOXC1 and FAM171A1 are direct targets of miR-9-3p, and RGMA is the direct target of miR-135b-3p

**Fig. 5:**
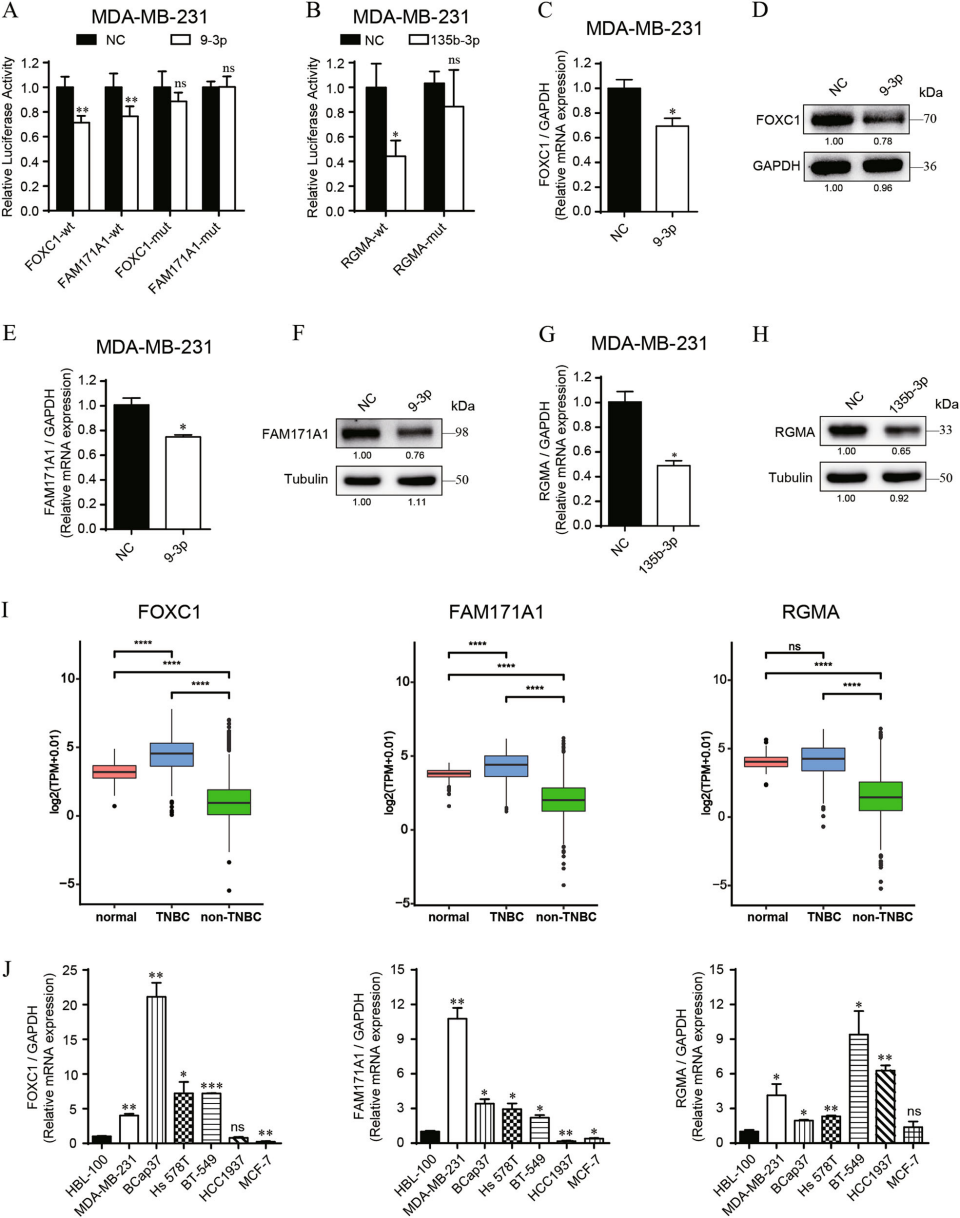
MiR-9-3p directly targets FOXC1/FAM171A1, and miR-135b-3p directly targets RGMA. **a**, **b** The dual luciferase assays in MDA-MB-231 cells confirmed that miR-9-3p directly targets FOXC1/FAM171A1, and miR-135b-3p directly targets RGMA. **c**–**h** The mRNA and protein levels of FOXC1, FAM171A1 and RGMA were measured in MDA-MB-231 with transfection of specific miRNA mimics. **i** The expression levels of FOXC1, FAM171A1 and RGMA were compared among normal (*n* = 113), TNBC (*n* = 115), non-TNBC (*n* = 973) samples form TCGA database. **j** The mRNA expressions of FOXC1, FAM171A1 and RGMA were measured in HBL-100 (normal), TNBC (MDA-MB-231, BCap37, Hs 578 T, BT-549, HCC1937) and non-TNBC (MCF-7) cell lines. Bars indicate the mean ± SD of three independent replicates. *P < 0.05, **P < 0.01, ***P < 0.001, ****P < 0.0001

To confirm the miRNA-gene targeting, dual-luciferase reporter assays were performed in MDA-MB-231 cells. The binding sites between miRNAs and 3′-UTRs of genes are shown in Fig. S4A. As a result, miR-9-3p downregulated the luciferase activities of wide-type 3′-UTRs of FOXC1 and FAM171A1 (Fig. 5a), whereas no reduction was shown with wide-type 3′-UTRs of BCL11A (Fig. S4B); miR-135b-3p exhibited significantly decreased luciferase activities of the wide-type 3′-UTRs of RGMA (Fig. 5b). Meanwhile, these two miRNAs could not downregulate the luciferase activities of mutant-type 3′-UTR reporters. Moreover, the mRNA and protein levels of FOXC1, FAM171A1 and RGMA were significantly downregulated in MDA-MB-231 cells (Fig. 5c–h) and BCap37 cells (Fig. S4C–H) with overexpression of upstream miRNAs. In addition, the expression levels of 4 hub genes were detected in BRCA cohorts in TCGA database. The results showed FOXC1, FAM171A1 and BCL11A were high expressed in TNBC samples but low expressed in non-TNBC samples, both compared with normal BRCA samples (Fig. 5i, Fig. S4I). However, RGMA only exhibited notable low expression in non-TNBC samples, but no significance showed in TNBC samples, compared with normal samples (Fig. 5i). Nevertheless, these 4 hub genes all showed notable higher expression in TNBC compared with non-TNBC cohort (Fig. 5i, Fig. S4I). Furthermore, the gene expressions were verified in normal (HBL-100), TNBC (MDA-MB-231, BCap37, Hs 578 T, BT-549, HCC1937) and non-TNBC (MCF-7) cell lines. The results showed 4 hub genes were significantly upregulated in most TNBC cell lines, compared to HBL-100 cells (Fig. 5j, Fig. S4J). Meanwhile, FOXC1, FAM171A1 and BCL11A were markedly downregulated in MCF-7 cells, compared to HBL-100 cells, but RGMA showed no significance (Fig. 5j, Fig. S4J). In conclusion, we proved that FOXC1 and FAM171A1 were two direct targets of miR-9-3p, and RGMA was the direct target of miR-135b-3p. Considering no targeting was shown between miR-9-3p and BCL11A, our further study mainly focused on the function of FOXC1, FAM171A1, and RGMA.

### The proliferation and migration of TNBC cells are suppressed by downregulation of FOXC1, FAM171A1 or RGMA

**Fig. 6:**
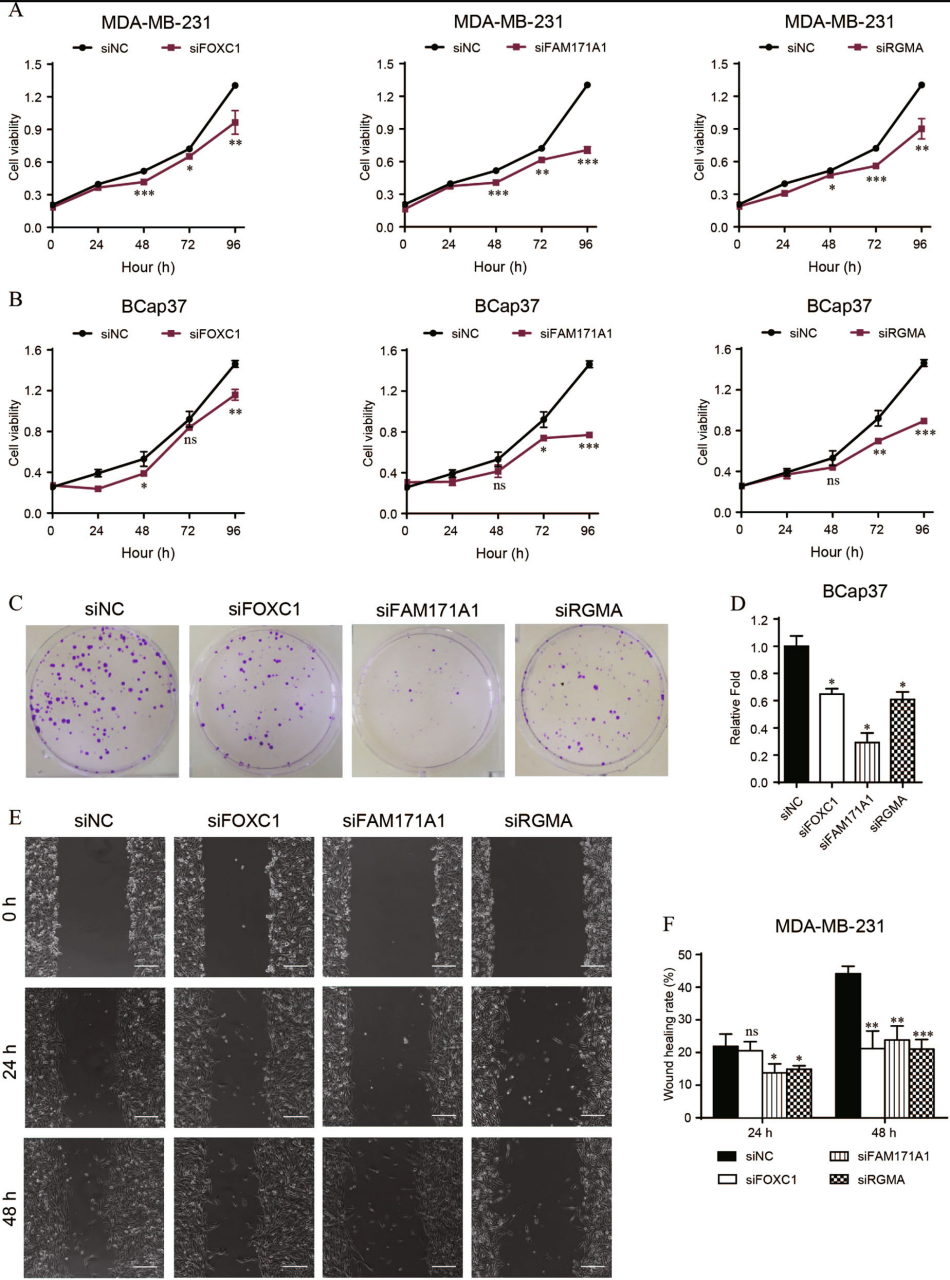
Downregulation of FOXC1, FAM171A1 or RGMA inhibits cell proliferation and migration of TNBC cell lines. **a**, **b** The cell growth curves of MDA-MB-231 and BCap37 cells with knockdown of FOXC1, FAM171A1 or RGMA. **c**, **d** The colony formation assays of BCap37 cells with downregulation of FOXC1, FAM171A1 or RGMA. **e**, **f** The wound healing rates of MDA-MB-231 cells with transfection of specific siRNAs at both 24 h and 48 h measurements. Scale bars: 200 μm. Bars indicate the mean ± SD of three independent replicates. *P < 0.05, **P < 0.01, ***P < 0.001

In view of high expression of FOXC1, FAM171A1 and RGMA in TNBC cell lines, we knockdown them in MDA-MB-231 and BCap37 cells by specific siRNAs for functional investigations (Table S3). The siRNAs efficiency was measured at mRNA and protein levels (Fig. S1F-H). Downregulation of FOXC1, FAM171A1 or RGMA was found to significantly inhibit cell growth and clonogenicity of TNBC cells by performing MTT cell viability assays (Fig. 6a, b) and colony formation assays (Fig. 6c, d). Moreover, downregulation of FAM171A1 or RGMA in MDA-MB-231 cells showed notable suppression of migration at both 24 h and 48 h measurements, whereas downregulation of FOXC1 functioned only at 48 h measurement (Fig. 6e, f).

## Discussion

Breast cancer is a heterogeneous disease with distinct histopathological feature and clinical behavior among subtypes. TNBC has attracted much attention as the most aggressive subtype, lacking of reliable biomarkers and efficient therapies. In current study, we aimed to find out special molecules associated only with the prognosis of TNBC rather than non-TNBC.

Some upfront studies have indicated that miRNA expression profiles are different in tumor specimens compared with peritumoral samples, and dysregulation of miRNAs correlates with progression and prognosis of tumors, including breast cancer^30^. Although miRNAs with small size, they have been reported to be more stable than mRNAs^31^, thus they can serve as ideal biomarkers for prognosis evaluation after pathologic biopsies^32^. In this study, by comprehensive analyses of large-scale miRNAs expression data from TCGA database, we successfully screened out 4 candidate DEmiRNAs (miR-135b-5p, miR-9-3p, miR-135b-3p, miR-455-5p) in TNBC but not in non-TNBC, both compared with normal samples. The survival analyses confirmed that high levels of miR-135b-5p, miR-9-3p and miR-135b-3p showed good prognosis, whereas high expression of miR-455-5p exhibited poor prognosis in TNBC. Nevertheless, there are two sets of miRNA expression data in TCGA database, classified by different sequencing methods: illumina-GA and illumina-hiseq. The analysis performed in our study did not simply merged these two datasets, but use the miRNA dataset sequenced by illumina-hiseq due to its larger number of miRNAs and samples.

Previously, miR-135b-5p has been well documented as a tumor suppressor^33–35^ or oncogene^36–38^ in breast cancer, but less is known about the expression and function of miR-135b-3p. In case that both 5p and 3p strands are functional and not degraded, despite targeting different mRNAs, they might cooperate temporally and result in a synergistic effect. For instance, miR-155-5p and miR-155-3p, by targeting respective mRNAs, cooperatively regulate the balance of interferon production^39^. In our screening, high levels of miR-135b-5p and miR-135b-3p both yielded better overall survival(OS) of TNBC cohort from TCGA database. In humans, the transcription of miR-9 ultimately give rise to two functional mature miRNAs, i.e. miR-9-5p and miR-9-3p^40^. Recent study reported that miR-9 could serve as a prognostic biomarker in TNBC, since its high expression was associated with poor prognosis of TNBC patients. However, the number of samples evaluated in this study was limited, and the mechanisms by which miR-9 influence prognosis of patients remained largely unclear^41^. In addition, miR-9 was proposed to act as both tumor suppressor and oncogenic roles in breast cancers^42,43^. As for miR-9-3p, although less intensively investigated in breast cancer, it has been identified as a tumor suppressor in claudin-low breast cancer cells^44^. Moreover, downregulation of miR-9-3p was reported to be related to worse clinical chemotherapy response in breast cancer^45^. However, the specific role of miR-9-3p in TNBC remains unknown. In this study, we identified miR-9-3p for the first time as a prognosis-related DEmiRNAs in TNBC. Likewise, the function of miR-455-5p in TNBC also remains to be elucidated. To the best of our knowledge, only one study indicated that miR-455-5p was differentially expressed between invasive and non-invasive breast cancer based on the analyses of archived FFPE tissues and laboratory-based barcoded cDNA library^46^. In other cancers, miR-455-5p has been reported as a potential oncogene for non-small cell lung cancer^47^, colon cancer^48^ and oral squamous cancer^49^. In our present study, we confirmed these 4 screened DEmiRNAs not only differentially expressed in TNBC cell lines compared to normal breast cell line, but also showed effective regulation on cell proliferation and migration.

Furthermore, we identified 4 hub genes for their highest correlation with TNBC, that is FOXC1, BCL11A, FAM171A1 and RGMA. Remarkably, our study verified that FOXC1 and FAM171A1 were two direct targets of miR-9-3p, and RGMA was the direct target of miR-135b-3p. In view of no targeting between miR-9-3p and BCL11A, we mainly focused on investigating the function of FOXC1, FAM171A1 and RGMA. FOXC1 was previously regarded as a participant in embryonic development^50^. Recently, a large body of literature have shown that FOXC1 plays a critical role in proliferation, metastasis, survival and chemosensitivity of TNBC, served as a potential therapeutic target^51–53^. Likewise, RGMA initially has been considered as an axon guidance molecule during embryogenesis^54^, but lately as a potential tumor suppressor in some cancers^55–57^. However, less is known about the explicit function of RGMA in TNBC. Last but not least, the newly discovered FAM171A1 has occurred in different settings of screening for gene expression or proteomics, also called Astroprincin(APCN) for its abundant expression in astrocytes^58^. Emerging evidences have suggested FAM171A1 is potentially overexpressed in TNBC^59–61^, but its biological function and significance remains poorly understood. Intriguingly, FOXC1, FAM171A1 and RGMA are all involved in brain development and tumorigenesis, that may explain why TNBC metastasize to brain more often. In current study, the expression levels of FOXC1, FAM171A1 and RGMA were compared among normal, TNBC and non-TNBC cohorts from TCGA database, and were measured in various cell lines. As a result, these 3 genes showed notable high expression in TNBC than non-TNBC samples of TCGA, which were largely consistent with their expression levels in TNBC and non-TNBC cell lines. More importantly, we confirmed that knockdown of FOXC1/FAM171A1/RGMA yielded inhibitory effects on cell proliferation and migration MDA-MB-231 and BCap37 cells, which was congruous with the function of respective upstream miRNAs (miR-9-3p/miR-135b-3p).

In summary, we identified 4 DEmiRNAs (miR-135b-5p, miR-9-3p, miR-135b-3p, miR-455-5p) which were significantly associated with TNBC prognosis, and 3 hub genes (FOXC1, FAM171A1, RGMA) with highly positive correlation with TNBC cohort. The experimental validations lay great emphasis on determining the functions and targeting relationships of these molecules in TNBC cell lines. However, further study is needed to uncover more precise behaviors and mechanisms of these TNBC-specific miRNAs and genes. Overall, we brought new insights that these identified molecules could potentially serve as prognostic biomarkers and therapeutic targets for TNBC.

## Supplementary information

Figure S1

Figure S2

Figure S3

Figure S4

Supplementary Figure Legends

Table S1

Table S2

Table S3
