# Supplementary figures and images for: Exploring specific prognostic biomarkers in triple-negative breast cancer
Source: Cell Death Dis. 2019 Oct 24;10(11):807. doi: 10.1038/s41419-019-2043-x (PMC6813359; doi:10.1038/s41419-019-2043-x)

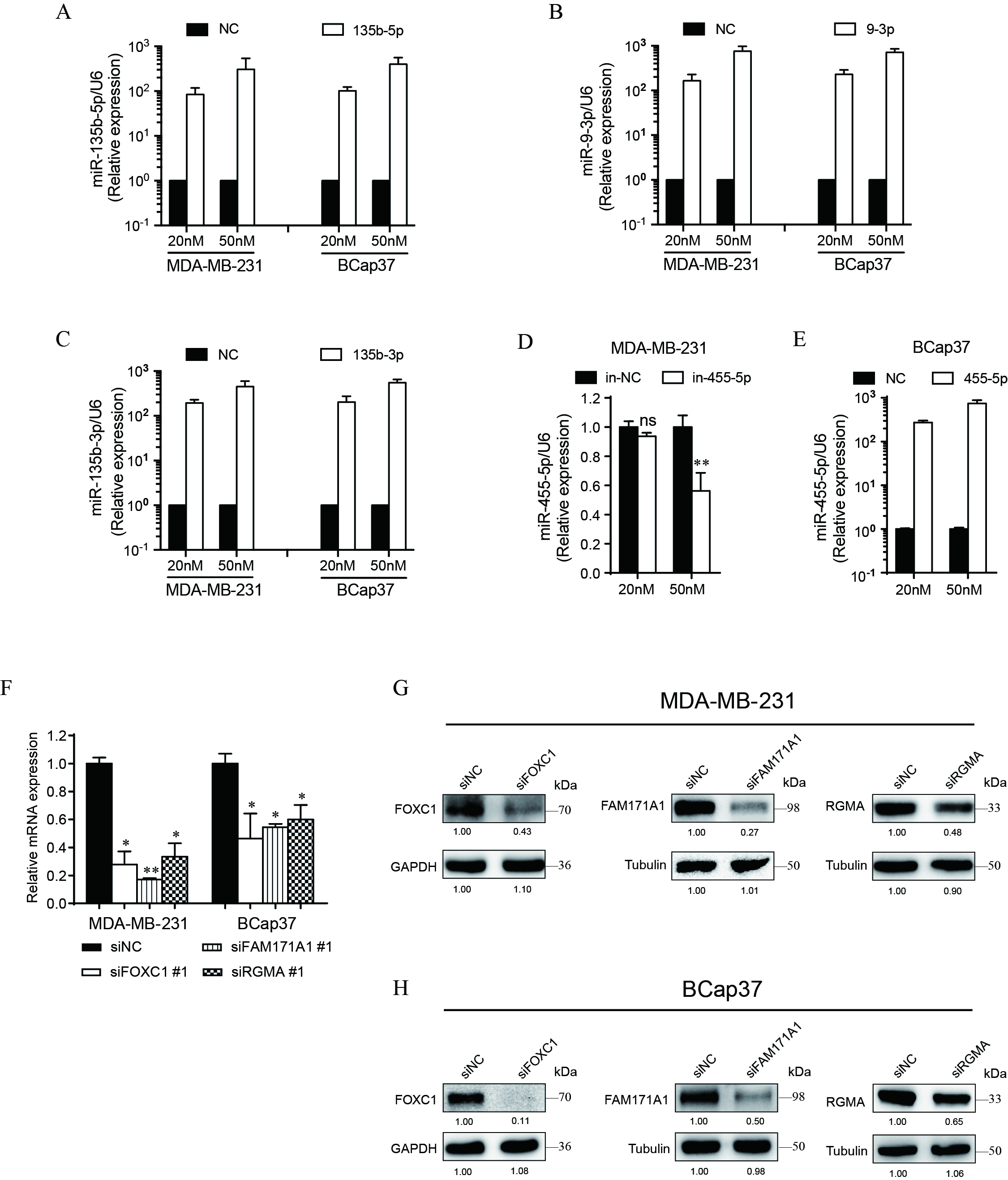

Supplement: Supplementary file 1 — Figure S1 [file 41419_2019_2043_MOESM1_ESM.jpg]

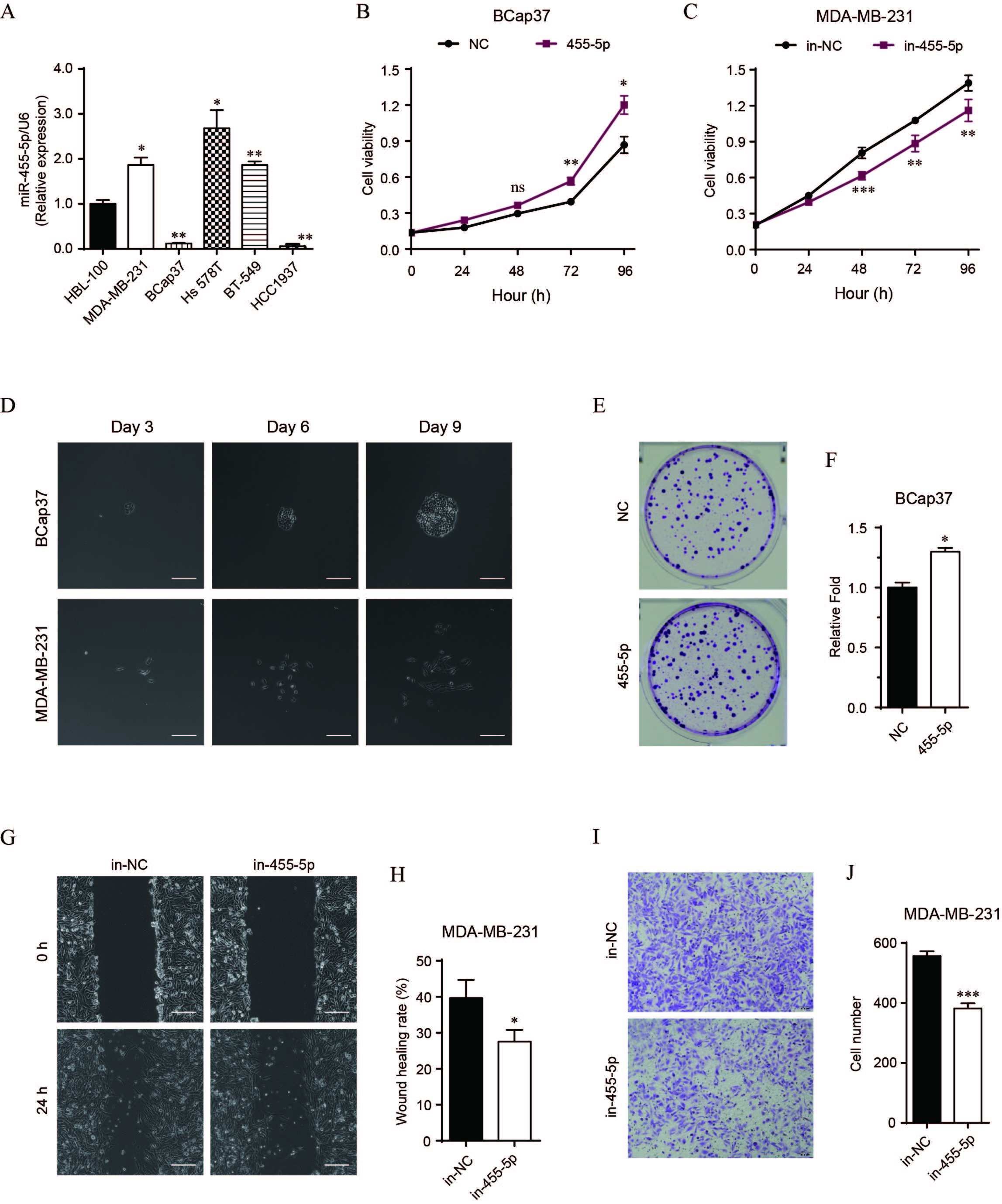

Supplement: Supplementary file 2 — Figure S2 [file 41419_2019_2043_MOESM2_ESM.jpg]

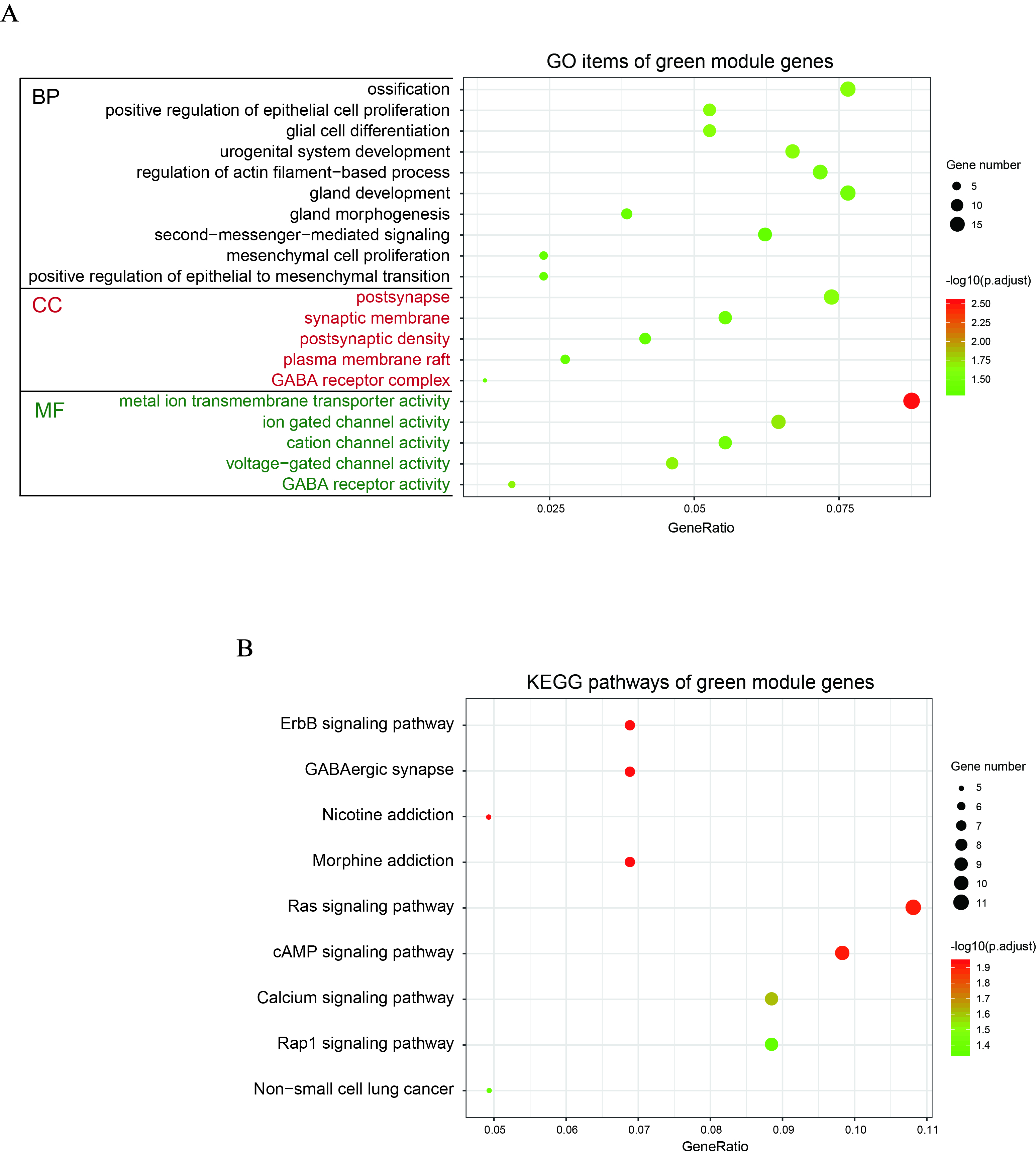

Supplement: Supplementary file 3 — Figure S3 [file 41419_2019_2043_MOESM3_ESM.jpg]

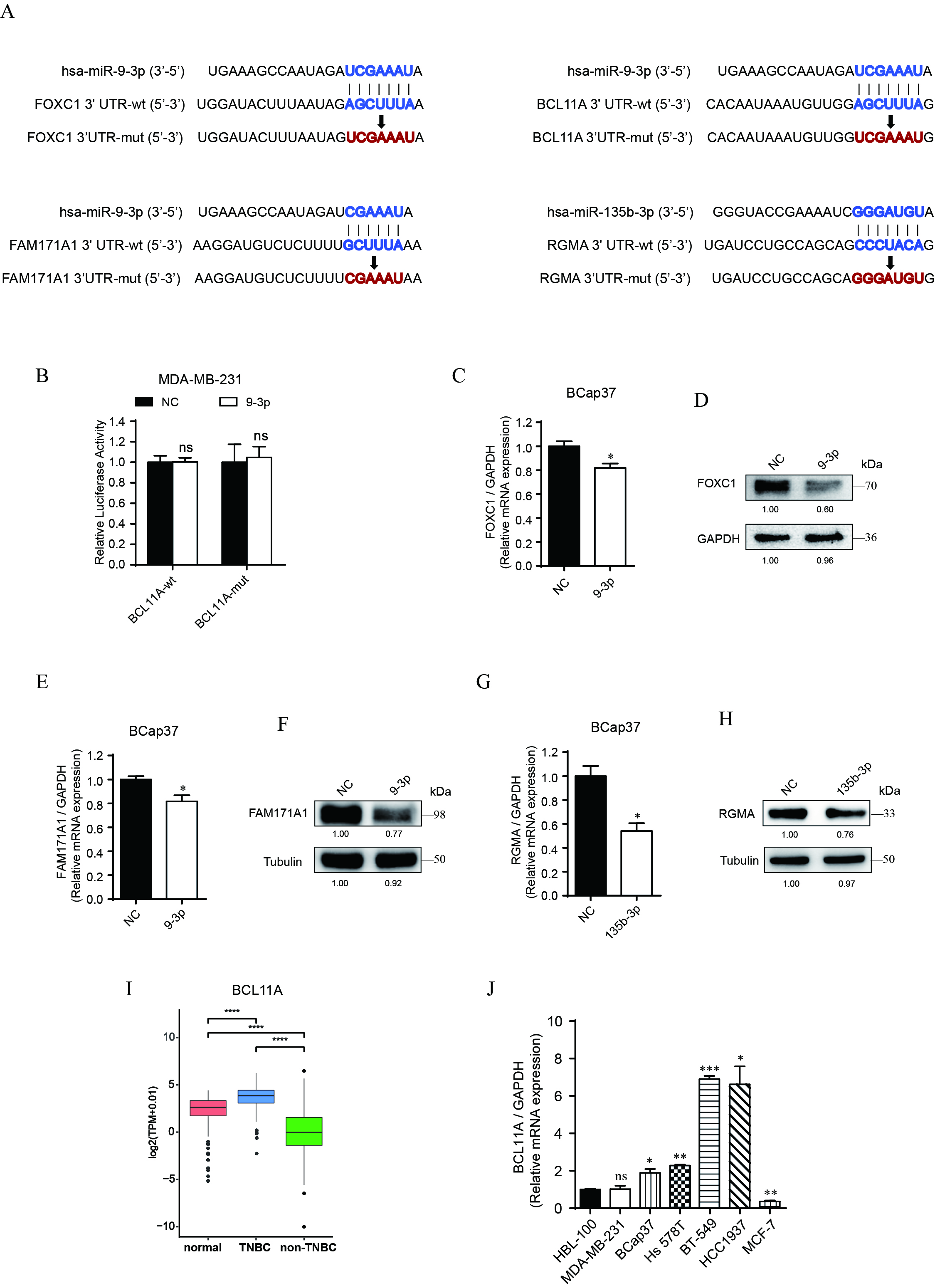

Supplement: Supplementary file 4 — Figure S4 [file 41419_2019_2043_MOESM4_ESM.jpg]
