# Supplementary material for: Exploring specific prognostic biomarkers in triple-negative breast cancer
Source: Cell Death Dis. 2019 Oct 24;10(11):807. doi: 10.1038/s41419-019-2043-x (PMC6813359; doi:10.1038/s41419-019-2043-x)
Supplement: Supplementary file 5 — Supplementary Figure Legends [file 41419_2019_2043_MOESM5_ESM.docx]

Supplementary Figure Legends

**Supplementary Figure 1**

**A-C** MDA-MB-231 and BCap37 cells were transfected with 20 or 50 nM of NC mimics (NC), miR-135b-5p mimics (135b-5p), miR-9-3p mimics (9-3p) or miR-135b-3p mimics (135b-3p). The expression levels of these 3 miRNAs were measured. **D, E** MDA-MB-231 cells were transfected with 20 or 50 nM of NC inhibitors (in-NC) or miR-455-5p inhibitors (in-455-5p), while BCap37 cells were transfected with 20 or 50 nM of NC mimics (NC) or miR-455-5p mimics (455-5p). The expression of miR-455-5p was measured. **F** MDA-MB-231 and BCap37 cells were transfected with 100 nM of NC siRNAs (siNC), FOXC1 siRNAs (siFOXC1), FAM171A1 siRNAs (siFAM171A1) or RGMA siRNAs (siRGMA). The expression levels of FOXC1, FAM171A1 and RGMA were measured. **G, H** Western Blotting. MDA-MB-231 and BCap37 cells were transfected with 100 nM of siNC, siFOXC1, siFAM171A1 or siRGMA. The protein levels of FOXC1, FAM171A1 and RGMA were measured. Bars indicate the mean ± SD of three independent replicates. *P < 0.05, **P < 0.01.

**Supplementary Figure 2**

**A** The expression levels of miR-455-5p were measured in 5 TNBC cell lines (MDA-MB-231, BCap37, Hs 578T, BT-549, HCC1937) and a normal breast cell line (HBL-100). **B** The proliferation was notably promoted in BCap37 cells transfected with miR-455-5p. **C** The proliferation was markedly suppressed in MDA-MB-231 cells transfected with in-455-5p. **D** BCap37 showed tight cell growth whereas MDA-MB-231 showed dispersive cell growth, thus BCap37 cells were more suitable for colony formation assays. Scale bars: 200 μm. **E, F** The colony formation assay was performed in BCap37 cells transfected with miR-455-5p. **G, H** The 24 h wound healing rates were measured in MDA-MB-231 cells transfected with in-455-5p. Scale bars: 200 μm. **I, J** The transwell migration assays were performed in MDA-MB-231 cells transfected with in-455-5p. Scale bars: 100 μm. Bars indicate the mean ± SD of three independent replicates. *P < 0.05, **P < 0.01, ***P < 0.001.

**Supplementary Figure 3**

**A** GO items of genes in the green module, including biological process (BP), cellular component (CC) and molecular function (MF). **B** KEGG enrichment analysis of genes in the green module.

**Supplementary Figure 4**

**A** The binding sites (blue) between miR-9-3p and 3’-UTRs of FOXC1/BCL11A/FAM171A1, miR-135b-3p and 3’-UTRs of RGMA in wide type (wt) reporter plasmids. The wt vectors were performed 6 or 7 bp mutation (red) at the binding sites, that is mutant type (mut) reporter plasmids. **B** The luciferase activity showed no reduction in MDA-MB-231 cells with co-transfection of miR-9-3p and wt/mut reporter plasmids. **C-H** The mRNA and protein levels of FOXC1, FAM171A1 and RGMA were measured in BCap37 cells with transfection of specific miRNAs. **I** The expression of BCL11A was compared among normal (n=113), TNBC (n=115), non-TNBC (n=973) samples form TCGA database. **J** The mRNA expression of BCL11A was measured in HBL-100 (normal), TNBC (MDA-MB-231, BCap37, Hs 578T, BT-549, HCC1937) and non-TNBC (MCF-7) cell lines. Bars indicate the mean ± SD of three independent replicates. *P < 0.05, **P < 0.01, ***P < 0.001, ****P < 0.0001.
