# Supplementary material for: Exploring specific prognostic biomarkers in triple-negative breast cancer
Source: Cell Death Dis. 2019 Oct 24;10(11):807. doi: 10.1038/s41419-019-2043-x (PMC6813359; doi:10.1038/s41419-019-2043-x)
Supplement: Supplementary file 7 — Table S2 [file 41419_2019_2043_MOESM7_ESM.docx]

Table S2. Sequences for primers used in this study

| **Name** | **Sequence** |
| --- | --- |
| **Primers for qRT-PCR**  RGMA-F  RGMA-R  FOXC1-F  FOXC1-R  FAM171A1-F  FAM171A1-R  BCL11A-F  BCL11A-R  GAPDH-F  GAPDH-R  **Primers for 3’UTR**  RGMA-F  RGMA-R  FOXC1-F  FOXC1-R  FAM171A1-F  FAM171A1-R  BCL11A-F  BCL11A-R  mut-RGMA-F  mut-RGMA-R  mut-FOXC1-F  mut-FOXC1-R  mut-FAM171A1-F  mut-FAM171A1 –R  mut-BCL11A-F  mut-BCL11A -R | CCAAGTACATCGGCACCACCATC  CTCCACAGCATTGACCACTTCCTC  CGCCACAACCTCTCGCTCAAC  TCCTTCTCCTCCTTGTCCTTCACC  CGTGGAACAGAGTGGTAGCGAATG  CAGTCCGTCTGGTCGTCTCCTC  AGATGAGCCCAGCAGCTACA  GGCTGGGAAGGACATTCTGC  TCTCCTCTGACTTCAACAGCGAC  CCCTGTTGCTGTAGCCAAATTC  TAGGCGATCGCTCGACTTCAACCTCTAATCTCTTCCCT  TTGCGGCCAGCGGCCAGGGACCAACTAAAACCAGCA  TAGGCGATCGCTCGACGCCAGAAAGTGTTCCCA  TTGCGGCCAGCGGCCATATCTTTTTACTAGCCTCAAAGCA  TAGGCGATCGCTCGACTCTTAACATCCAAGCCTTTTAATG  TTGCGGCCAGCGGCCTCCAGAGTTAATACGTAACCG  TAGGCGATCGCTCGACCCACATTGGAACAGTGA  TTGCGGCCAGCGGCCATATTGCTATGATTTATTCCCAAGT  GCAGGGGATGTGCTCCACACCCTACCCACCCAT  GAGCACATCCCCTGCTGGCAGGATCACCCTCCA  ACTTTAATAGTCGAAATATTATTACGAAAAAAGAT  TCGTAATAATATTTCGACTATTAAAGTATCCAGAC  TGTCTCTTTTCGAAATAACTGCATGCTGCCAAGT  GCATGCAGTTATTTCGAAAAGAGACATCCTTTAA  ACACAATAAATGTTGGTCGAAAT  CAAGCAAACACCACATTTCGACCAA |
