# Supplementary material for: Exploring specific prognostic biomarkers in triple-negative breast cancer
Source: Cell Death Dis. 2019 Oct 24;10(11):807. doi: 10.1038/s41419-019-2043-x (PMC6813359; doi:10.1038/s41419-019-2043-x)
Supplement: Supplementary file 8 — Table S3 [file 41419_2019_2043_MOESM8_ESM.docx]

Table S3. Sequences for siRNAs used in this study

| Name | Sequence |
| --- | --- |
| siRGMA# 1(sense)  siRGMA# 1(antisense)  siRGMA# 2(sense)  siRGMA # 2 (antisense)  siFOXC1# 1(sense)  siFOXC1# 1 (antisense)  siFOXC1# 2(sense)  siFOXC1# 2 (antisense)  siFAM171A1# 1 (sense)  siFAM171A1# 1 (antisense)  siFAM171A1# 2 (sense)  siFAM171A1# 2 (antisense) | GCCAGCACAACUGCUCCAATT  UUGGAGCAGUUGUGCUGGCTT  CCAUUACGAGAAGAGCUUUTT  AAAGCUCUUCUCGUAAUGGTT  GGGAAUAGUAGCUGUCAAATT  UUUGACAGCUACUAUUCCCTT  GGCCAGAUAUGCACAGAUATT  UAUCUGUGCAUAUCUGGCCTT  CCACGUCCAUGUCACACAUTT  AUGUGUGACAUGGACGUGGTT  GGAUUAGACGGAAAUGGAATT  UUCCAUUUCCGUCUAAUCCTT |
